# Effectiveness of exercise interventions to improve long-term outcomes in people living with mild cognitive impairment: a systematic review and meta-analysis

**DOI:** 10.1038/s41598-023-44771-7

**Published:** 2023-10-23

**Authors:** Mirjam Dieckelmann, Ana I. González-González, Winfried Banzer, Andrea Berghold, Klaus Jeitler, Johannes Pantel, Gudrun Pregartner, Arthur Schall, Valentina A. Tesky, Andrea Siebenhofer

**Affiliations:** 1 Institute of General Practice, Goethe University Frankfurt, Theodor-Stern-Kai 7, 60590 Frankfurt, Germany; 2 Red de Investigación en Cronicidad, Atención Primaria y Promoción de la Salud (RICAPPS), Madrid, Spain; 3 Institute for Occupational, Social and Environmental Medicine, Goethe University Frankfurt, Frankfurt, Germany; 4 Institute for Medical Informatics, Statistics and Documentation, Medical University of Graz, Graz, Austria; 5 Institute of General Practice and Evidence-Based Health Services Research, Medical University of Graz, Graz, Austria

**Keywords:** Disease prevention, Neurological disorders

## Abstract

Although exercise guidelines now recommend exercise for patients with MCI, the long-term effects of exercise in patients with MCI has not been reviewed systematically. The aim was to assess (1) the effectiveness of exercise and physical activity (EXPA) interventions in improving long-term patient-relevant cognitive and non-cognitive outcomes in people with mild cognitive impairment, (2) how well the included trials reported details of the intervention, and (3) the extent to which reported endpoints were in line with patient preferences that were assessed in patient workshops. Following PRISMA guidelines, we performed a systematic review and meta-analysis including randomized controlled trials. A total of ten studies were included after searching in six electronic sources from 1995 onwards. There is a trend that 6 + -month EXPA interventions improve global cognition 12 months after initiation. Evidence on long-term effects of EXPA interventions on non-cognitive health outcomes could not be meaningfully pooled and the individual studies reported mixed results. Workshop participants considered freedom from pain and stress, mood, motivation and self-efficacy to be important, but these outcomes were rarely addressed. Too little information is available on intervention details for EXPA programs to be replicated and confidently recommended for patients with MCI. PROSPERO registration in December, 2021 (CRD42021287166).

## Introduction

Mild cognitive impairment (MCI) is a syndrome characterized by cognitive deficits that do not significantly interfere with activities of daily living, and therefore does not fulfil the criteria for dementia^1^. However, people affected by MCI are at greater risk of dementia, with a yearly conversion rate of 15 to 41%^2^. In the absence of a curative therapy, persons with progressive MCI face the burden of increasing dysfunction in both cognitive ability and non-cognitive health, including physical functioning and fitness, quality of life as well as social and psychological wellbeing^3–5^. It also commonly affects the well-being of close relatives^6^.

Animal studies consistently report improved neuroplasticity and anti-inflammatory effects in physically active mice^7^. However, these biochemical effects of exercise on brain structure and brain function are lesser understood in humans. It is discussed that exercise induces neuroprotection and neurorestoration, enhances cognitive function and delays cognitive decline by mitigating cardiovascular risk factors in people with MCI to improve cognitive health and brain health^8–10^. Apart from the effect of exercise on cognition, functional independence and psychosocial health is shown to be improved in physically active people with MCI^11^.

A growing body of literature reports that exercise and physical activity (EXPA) can slow down the onset of dementia and prevent problems associated with it^1,12,13^. However, results from the literature are inconsistent and the effect of exercise or physical activity in MCI is not yet clear. For example, an umbrella review of EXPA interventions reports positive effects on global cognition, executive functions, delayed recall, and speed of processing among people with mild cognitive impairment and dementia, whereas EXPA had no effect on verbal fluency, immediate recall and attention^14^–the latter found to be effective in another umbrella review including interventional and observational data, however^15^. Both studies note that the evidence certainty was low-to-moderate. Most evidence stem from EXPA interventions of durations up to 6 months^13^ and we are not aware that long-term interventions have been reviewed separately.

Therefore, the aim of our study was to systematically review randomized controlled trials (RCTs) to assess the effectiveness of EXPA interventions in improving long-term patient-relevant cognitive and non-cognitive outcomes in people with MCI. Secondary research questions were whether the trials reported details of the intervention well enough for healthcare professionals to be able to prescribe the same exercise and physical activity for people with MCI in clinical practice, and the extent to which the reported endpoints reflected patient preferences.

## Methods

### Protocol and registration

We have described the methodology in more detail in a study protocol^16^ and have registered this study in PROSPERO (registration no. CRD42021287166). The present report follows the Preferred Reporting System Items for Systematic Review and Meta-Analysis (PRISMA) checklist^17^.

### Design

We performed a systematic review and meta-analysis of RCTs utilizing EXPA interventions to improve the knowledge about long-term patient-relevant cognitive and non-cognitive outcomes in people living with MCI.

### Eligibility criteria

#### Types of studies

We included RCTs that allowed the separate analysis of an exercise or physical activity intervention and compared it with any form of placebo, or an active (but physically inactive) control group. No restriction on study setting was applied. Studies were included if they were published in German, Spanish or English.

#### Participants

Studies had to report on adult individuals (50 + years) that had been diagnosed with MCI based on commonly applied criteria^18,19^, or on the study authors’ own definition as long as it involved a sound diagnosis by a neuropsychiatrist. Studies including participants whose diagnosis was not based on both subjective and objective cognitive impairment, or that included asymptomatic participants at increased risk of dementia, were not eligible. Healthy populations and populations with a diagnosis of dementia or cognitive impairment caused by traumatic injury or psychiatric disorders such as major depression were also excluded.

#### Interventions

We considered any intervention in which exercise or physical activity, defined as any form of structured exercise, recreational activity or bodily movement that results in elevated energy expenditure^20^, was the only intervention. The intervention also had to last 24 weeks or more, independent of type, intensity, volume, frequency, session duration, delivery mode and setting.

#### Comparators

Any well-designed placebo treatment such as sham exercise (i.e. stretching, toning, face, or finger exercise), or active (but physically inactive) control group (i.e. social visits and educational sessions) was considered acceptable. Comparisons between different exercise stimuli, as well as comparators of multimodal interventions such as combined exercise and cognitive training, were considered ineligible.

#### Outcomes

Since exercise regimens in real-life are to be maintained and initiated by patients themselves, we sought to understand if EXPA is effective in improving health of patients with MCI in the long run. Therefore, we extracted not only brain measures and cognitive outcomes, but also behavioral and motivational outcomes that were measured at least 48 weeks after the intervention began. Specifically, outcomes of interest were incidence of dementia or neuropsychiatric symptoms, global cognition, domain-specific cognition, (instrumental) activities of daily living, (health-related) quality of life, health care utilization, caregiver outcomes, psychosocial functioning, physical functioning, motivational parameters, neurobiological outcomes, and pain. We also included compliance parameters and the incidence of adverse events.

### Data sources and search strategy

Five electronic databases (MEDLINE, Embase, PsycINFO, SPORTDiscus and Cochrane’s Central Register of Controlled Trials CENTRAL) were searched from 1995 onwards to November/December 2021. A combination of Medical Subject Headings (MESH) and free-text terms was used for participants (e.g. cognitive dysfunction, cognition disorders, mild cognitive impairment, mild neurocognitive disorder), intervention (e.g. exercise, physical activity, sports), and study type (e.g. randomized controlled trial). The complete search strategy with exact search dates was published elsewhere^16^.

Furthermore, we hand-searched the Cochrane Dementia and Cognitive Improvement Group Specialized Register (ALOIS) for all results in the diagnosis category “MCI” and the intervention category “exercise or physical activity”. Finally, we performed a search of the Cochrane Database of Systematic Reviews (CDSR) to retrieve primary studies from the screening references of relevant systematic reviews. The authors were contacted when clinical trial registration entries were identified without a corresponding full-text publication.

### Study selection

Bibliographic details of all identified references were uploaded into COVIDENCE (4) and duplicates were removed. Title and abstract screening and eligibility assessment of full-texts by two independent reviewers (MD, AIG), respectively. Any discrepancy was solved through discussion and consensus, or with the help of a third reviewer (AS). No automation tools were used when selecting studies.

### Data extraction

The extracted data included study source, country, setting, design, duration (weeks), number of participants, distribution of sex, age, educational level (years), global cognition assessed using the Mini-Mental State Examination (MMSE) test^21^, fitness level, type of intervention and comparator, as well as frequency, duration and intensity of the exercise, and all eligible outcomes (see “Outcomes”).

If studies reported multiple test measures for a particular outcome parameter, all data were extracted. When available, 18-month follow-up results were extracted along with 12-month results. Some studies reported change scores and others reported baseline and final values. We extracted all the reported information and the respective group sizes. While some studies reported on completers, others reported on intention to treat (ITT) populations. We extracted all available information but treated completers preferentially in the analysis for the sake of homogeneity. All missing data was labelled “not reported” within the extraction sheet. The raw data extraction table was published in the Zenodo repository for open access^22^.

### Study risk of bias assessment and reporting quality of EXPA intervention details

Two reviewers (MD, AIG) assessed risk of bias (RoB) using the Cochrane Risk of Bias tool (RoB 1.0)^23^ in accordance with the Cochrane Handbook for Systematic Reviews of Interventions^24^. We used robvis to create RoB plots^25^. Additionally, one reviewer (MD) used the Consensus on Exercise Reporting Template (CERT) to assess the reporting quality of the EXPA interventions^26^.

### Synthesis methods

Some homogeneity between the studies was achieved by the use of narrow inclusion criteria regarding the control group design, and similarities in the duration of the intervention and the MCI criteria. Although eligibility criteria concerning settings and modes of intervention were rather broad, meta-analysis was only performed if a sport scientist (MD) categorized the intervention as structured exercise, recreational activity, or bodily movement resulting in elevated energy expenditure^20^.

Since all outcomes of interest were continuous, we used mean change scores from baseline to 12 (or 18) months as an effect measure, and calculated the change scores for studies that did not report them directly. If studies reported only standard errors, interquartile ranges or confidence intervals, SDs were calculated according to the Cochrane Handbook^24^. The design effect was taken into account when calculating the effective population size of included cluster-RCTs^27^.

A quantitative synthesis using random-effects meta-analyses was conducted if outcomes were deemed suitable to pool effect sizes. Advice on which outcome scales could be meaningfully pooled was provided by a psychologist (VT). Forest plots are presented for the mean differences in change scores between the study groups, along with 95% confidence intervals (CI). Higgins’ I^2^ was used to quantify outcome heterogeneity.

For cognitive outcomes, a number of different cognitive tests were analyzed separately. However, when a particular cognitive test involved sub-tests, they were considered together (for example, for verbal fluency, we combined categorical and lexical verbal fluency tests). As they all measure response inhibition, Stroop tests were combined in a meta-analysis that included test scenario SCWT-Abridged I, II or III, along with subtractions (i.e. SCWT II-III). Some outcomes had to be multiplied with − 1 (ADAS-COG, CDR-SOB, SCWT, TIADL, BAYER-ADL, Gait Speed, CSDD, BDI) so that higher scores reflected “better” results in all tests.

Where a formal meta-analysis could not be meaningfully conducted, we display data using forests plots but with no pooled effect measure. Data on secondary study objectives, CERT-outcomes and patient preferences, are presented in tabular form and synthesized narratively. Where meta-analysis was performed, we pooled all studies regardless of their RoB or the reporting quality of intervention details assessed by CERT. No subgroup and sensitivity analyses were performed. R version 4.2.1 was used for the statistical analyses.

### Assessment of patient preferences

In order to assess the extent to which outcomes reported in the included studies reflect patient preferences, two patient workshops were conducted with the aim of understanding which outcomes are meaningful to patients with MCI. Therefore, patients with amnestic MCI were recruited from a pool of patients who had participated in previous intervention studies conducted by the working group of gerontology and who had given their consent to be contacted for further studies or projects. Participants were informed about the overall aim of the systematic review project in an information letter and were notified that participants must not be physically active themselves in order to participate. A total of eight people with MCI gave their consent to participate in a workshop designed to learn about which outcome measures were viewed important. We conducted two workshops of four participants each. The duration of each workshop was 120 min and focused around the questions “which improvements do you believe can be achieved through physical activity and exercise in your personal life?” and “which of these improvements are more or less important to you in your everyday life?”. The conversations were audio-recorded and then transcribed verbatim. Data was analyzed using thematic analysis. A coding schema was developed using deductive categories of outcomes reported in the protocol^16^ as well as inductive categories emerging from the transcribed material.

## Results

**Figure 1:**
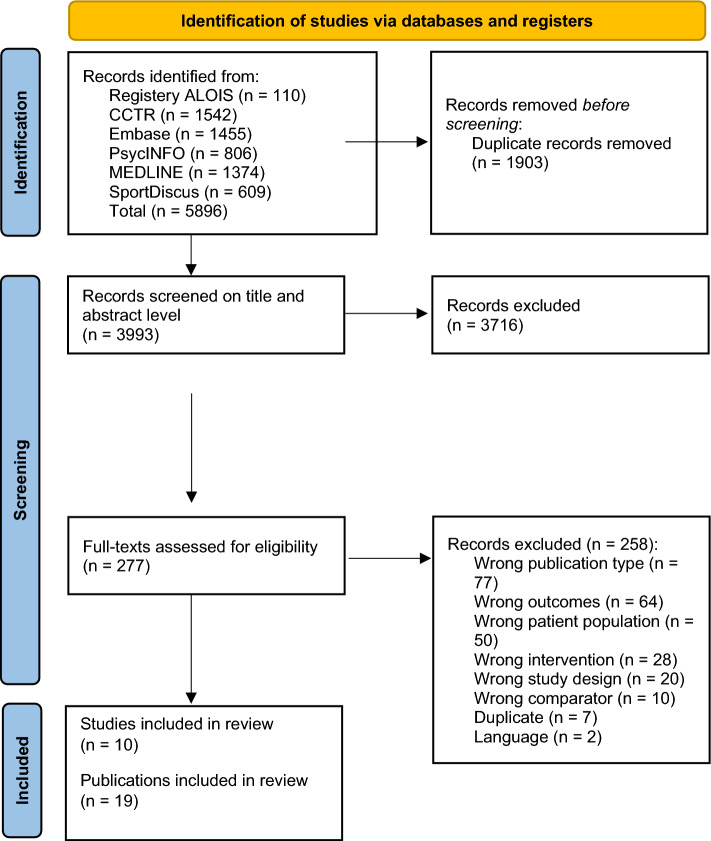
PRISMA flow diagram for systematic reviews^30^.

After screening 3993 records (after deduplication), 19 publications were included in the systematic review (Fig. 1). These 19 publications could be assigned to 10 studies, since multiple publications existed for most trials. None of the included publications was a language copy. We chose the unit of studies (not publications) to report frequencies. Most studies were excluded because they were not full-length publications based on an RCT, the intervention was shorter than 6 months, or the outcomes were assessed after 6 months without further follow up. Some studies would have been eligible from a design and outcome assessment perspective, but the studied population was either mixed (i.e. Sinclair et al.^28^), or the choice of participants was based only on MMSE scores (i.e. Varela et al.^29^).

The characteristics of the included studies are summarized in Table 1 and Supplementary Table 1. Overall, 10 RCTs involving 1741 adults with MCI (64% female, mean age range 65–78) were included. Three studies^31–38^ were from North America., two from Australia^39–41^, two from Europe^42–44^, two from China^45–47^ and one from Japan^48,49^. The recruitment setting was generally community-based (50%), with most populations stemming from the outpatient clinic of a hospital (50%), followed by long-term care centers (30%). The majority of interventions lasted 12 months (80%) and employed aerobic exercises of moderate intensity with a mean overall exercise volume of 79 ± 32 h. The EXPA setting was mostly supervised training in groups, followed by a mix of group-based sessions and unsupervised home sessions. Six studies reported that training was tailored to the individual, however only four provided a detailed description of how tailoring was achieved (i.e. individualized intensity according to the participants aerobic capacity, individualized progression of exercises according to the participant’s exercise response). On average, participants were not regularly active before beginning the EXPA interventions, had a mean education of six years and a mean MMSE of 26. Included interventions were mostly tested against control groups that implemented stretching and toning activities that did not meet the criteria for physical activity^20^.

**Table 1:** Descriptive summary of included studies.

Study characteristics	Total (n = 10)^†^
**Geographical location**	
North America	3 (30)
Europe	2 (20)
Australia	2 (20)
Asia	3 (30)
Setting*	
Community-based	5 (50)
Outpatient (hospital)	5 (50)
Long-term care centers/nursing homes	3 (30)
**Study design**	
Parallel arms	6 (60)
Cluster	2 (20)
2 × 2 factorial design	2 (20)
**Duration**	
12 months	8 (80)
18 months	2 (20)
**Population characteristics**	
Sample size at baseline (range)	20–555
Mean age (range)	65–78
Percent male participants (range)	22–70
Years of education (range)	3–16
Global cognition—MMSE, n = 1386, mean score (SD)	26 (2)
**Fitness level at baseline**	
Majority not regularly active/sedentary	5 (50)
Majority active	2 (20)
Not reported	3 (30)
Intervention design*	
Intervention length 12 months	6 (60)
Intervention length 6 months	4 (40)
Aerobic (walking)	5 (50)
Multicomponent exercise	2 (20)
Resistance training	1 (10)
Tai Chi	1 (10)
Interactive video gaming	1 (10)
Exercise frequency in times/week (range)	1–4
Duration in minutes/day (range)	25–90
Overall exercise volume–mean min/d × d/wk × wks (SD)	4722 (1910)
Moderate intensity (as reported)	6 (60)
High intensity (as reported)	2 (20)
Progressive intensity	1 (10)
Not reported	1 (10)
Control group design*	
Stretching and toning/low-intensity activity	5 (50)
Educational material/sessions	4 (40)
Social gathering	2 (20)

^†^n (%) or otherwise indicated, *studies may be included in more than one category.

**Figure 2:**
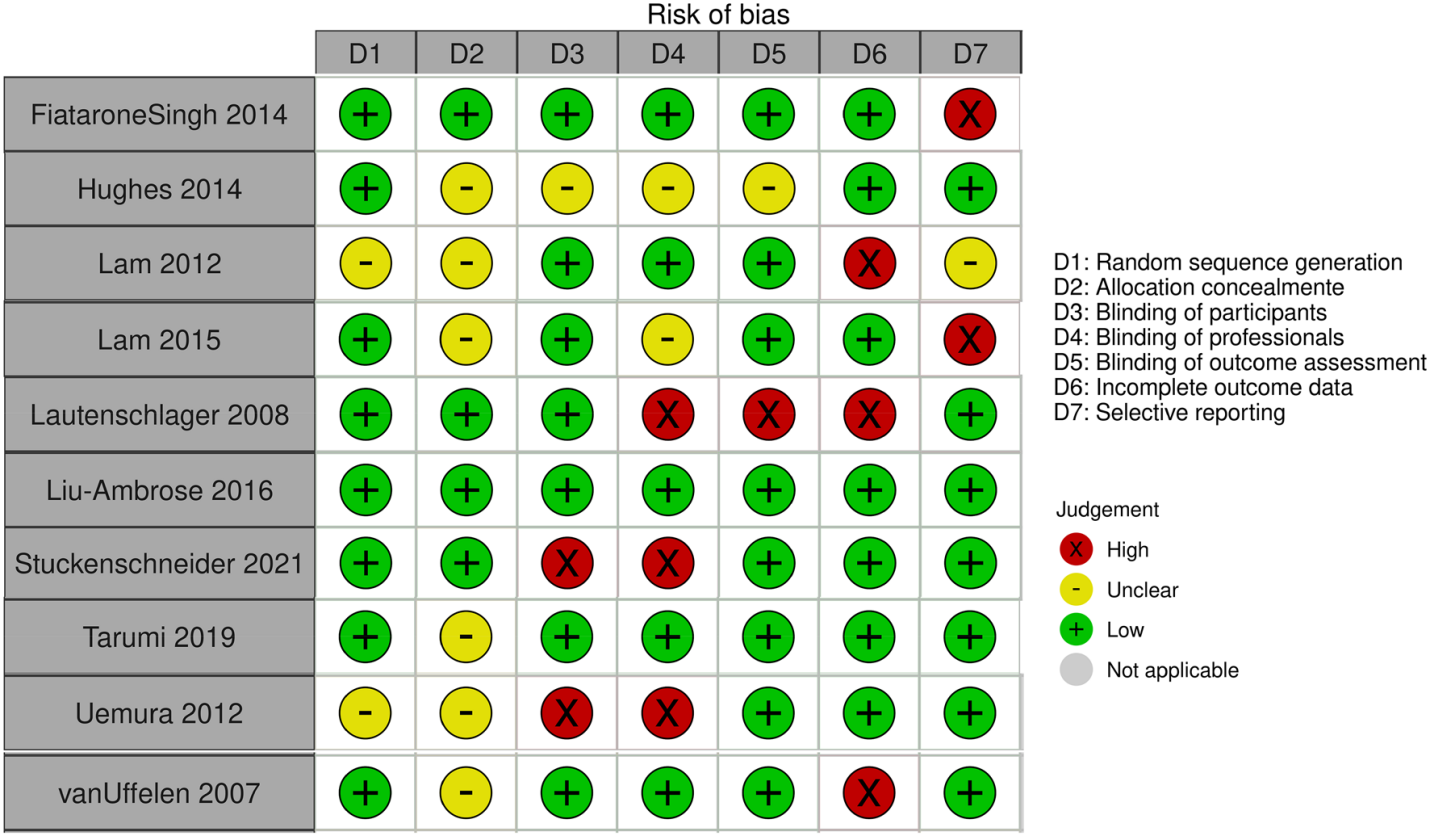
Risk of bias in the included studies.

Figure 2 summarizes the RoB in the included studies. All but two studies^46–49^ described the methodology for random sequence generation, but only four^32,33,39–42^ reported allocation concealment. Selection bias was unclear for the remaining studies. In five studies no performance bias could be detected^32–38,40,41,43,44,46,47^ and was high in two studies because participants were unblinded^42,48,49^, in a further three because healthcare professionals were unblinded^39,42,48,49^ and unclear for two studies^31,45^. Detection bias was high in one study^39^ and unclear in another^31^. The risk of attrition bias was high in three studies^39,43,44,46,47^ because of missing data. Reporting bias was high in two studies^40,41,45^ and unclear in one study^46,47^.

### Progression to dementia and neuropsychiatric symptoms 

Progression to dementia was evaluated by one study with mostly high and unclear RoB^46,47^. Although a trend towards lower risk development was seen in the ITT analysis, the differences between 12 months of Tai Chi and a stretching and toning group were not significant. The authors also used the Neuropsychiatric Inventory (NPI)^50^ to assess neuropsychiatric symptoms but found no significant differences between Tai Chi and Placebo Tai Chi.

### Global cognitive function 

Global cognitive function was measured in nine studies. Five moderate quality studies^32,39–41,45–47^ used the Alzheimer’s disease assessment scale–cognitive subscale (ADAS-Cog)^51^ to evaluate global cognitive status, three moderate quality studies^39,45–47^ used the clinical dementia rating sum of boxes (CDR SOB)^52^, one study with unclear RoB^31^ used the computerized assessment of mild cognitive impairment (CAMCI)^53^, four moderate quality studies^43–48^ the MMSE^21^, and two studies with high RoB arising from blinding procedures and selective reporting^41,42^ used an individual composite score for global cognition consisting of various domain-specific cognition tests.

The majority of these measures indicated that improvement in global cognition after 12 months was greater in the EXPA group than the sham-exercise group. Due to potential increase of detection bias because of the use of different procedures to detect global function no combined pooling of all studies were made. Analysing the studies assessing the outcome with the CDR SOB showed significant improvement in favour of the EXPA intervention in two of the three studies – an effect that was not obvious in any of the other tests of global cognition.

**Figure 3:**
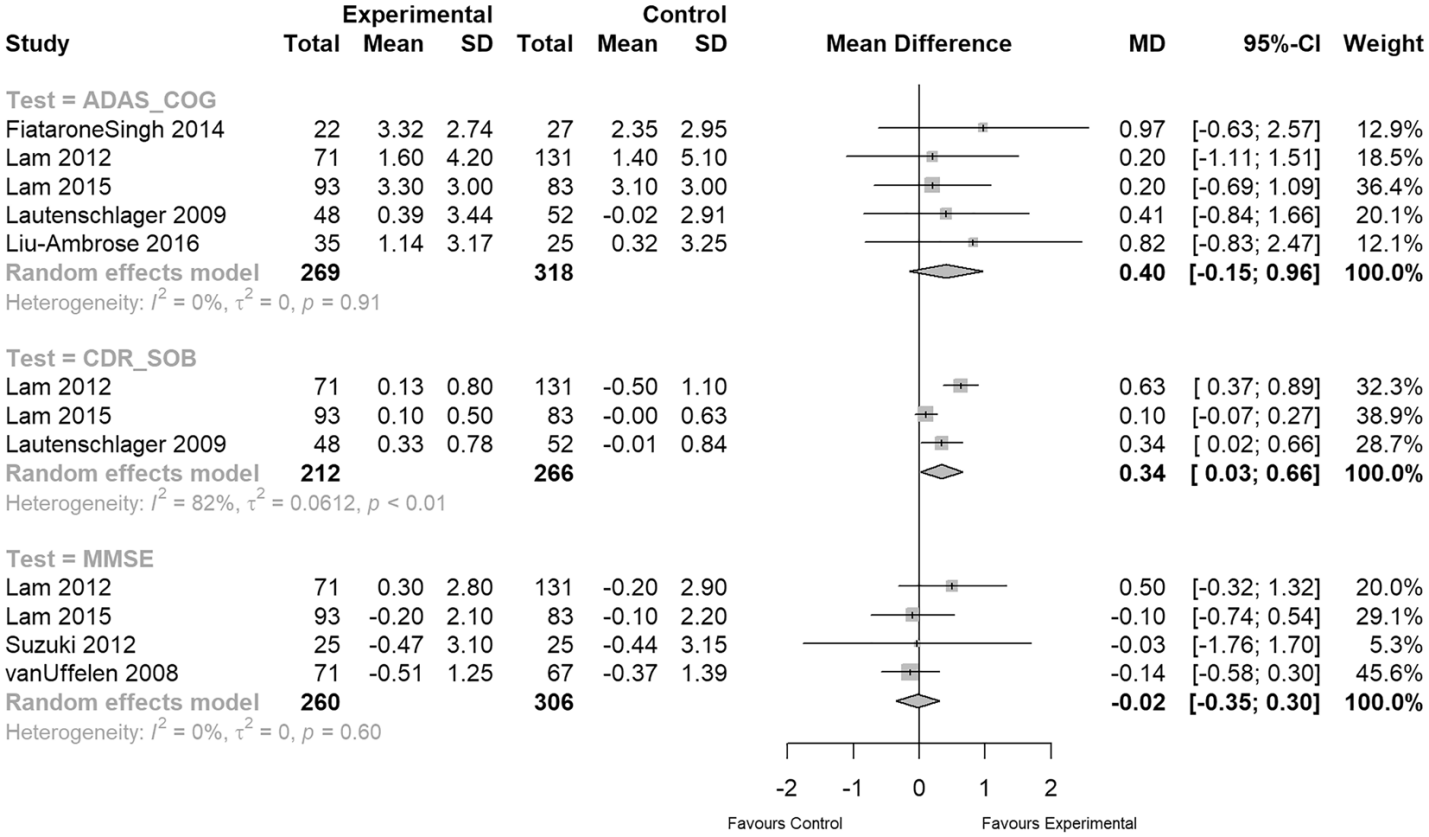
Meta-analyses of global cognition (ADAS-COG and CDR-SOB outcomes multiplied with − 1 so that higher scores reflect “better” results).

We performed a meta-analysis for ADAS-Cog (n = 587, MD = 0.40, 95% CI − 0.15 to 0.96), MMSE (n = 566, MD = – 0.02, 95% CI − 0.35 to 0.30) and CDR SOB measures, as shown in Fig. 3. The results show that participants in the EXPA group only showed significantly improved global cognitive ability in their CDR SOB scores (n = 478, MD = 0.34, 95% CI 0.03 to 0.66, p < 0.01, I^2^ = 82%; Fig. 3), however with considerable heterogeneity.

### Executive functions

Executive domain was assessed in all studies with overall moderate quality. They used the Matrices and Similarities subtests of the Wechsler Adult Intelligence Scale, Third Edition (WAIS-III)^54^, as well as categorical and lexical verbal fluency tests. The latter included the Controlled oral words association test (COWAT)^55^, Kaplan executive function system (KEFS)^56^, Cambridge contextual reading test^57^, executive interview (EXIT25)^58^, stroop colour and word test (SCWT)^59^, trail making test (TMT)^60^ and individual composite scores.

**Figure 4:**
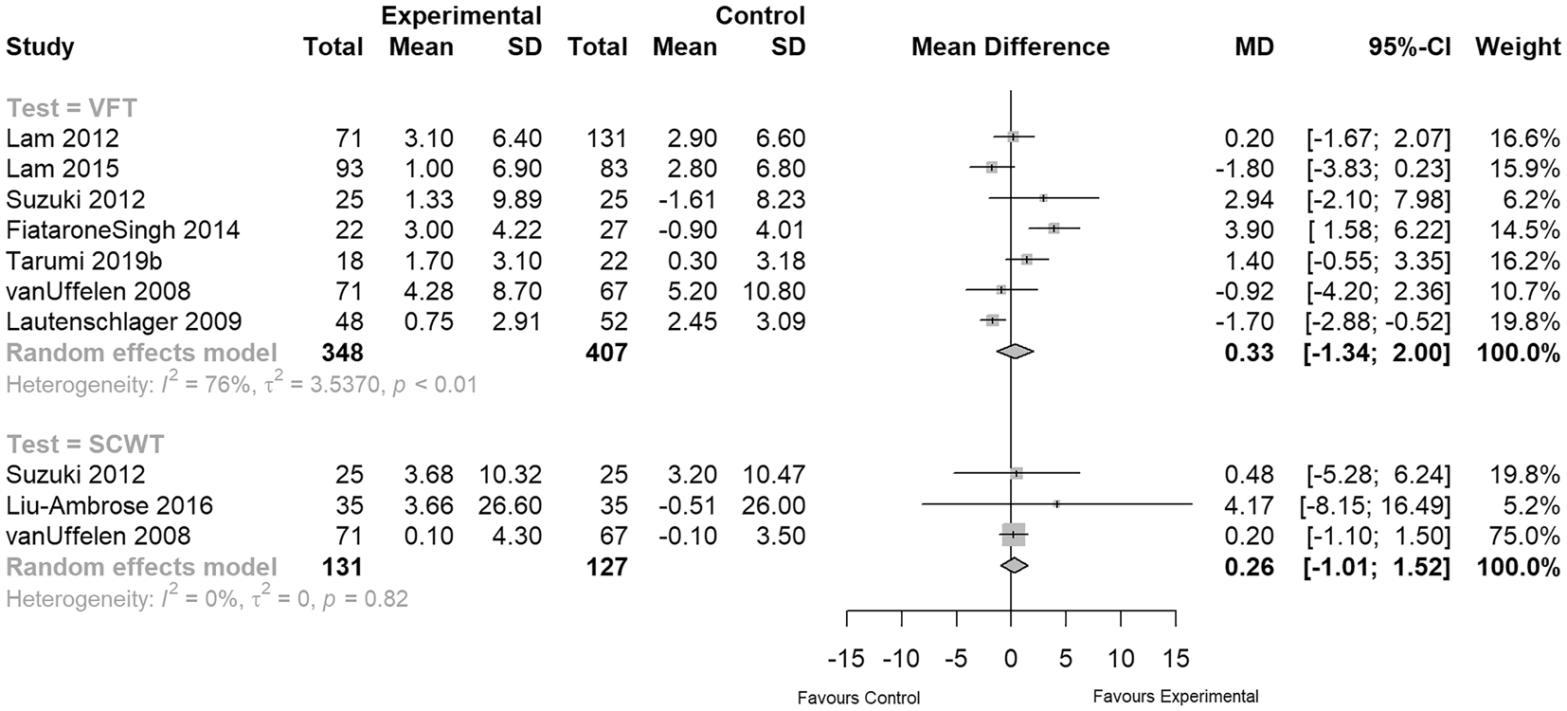
Meta-analyses of executive function (SCWT outcomes multiplied with − 1 so that higher scores reflect “better” results).

We were able to perform meta-analyses including data from seven studies for the verbal fluency tests by including both verbal and lexical test scenarios (VFT), as well as three studies for SCWT (see Fig. 4). After 12 months, VFT (n = 755, MD = 0.33, 95% CI − 1.34 to 2.00; Fig. 4) and SCWT (n = 258, MD = 0.26, 95% CI − 1.01 to 1.52; Fig. 4) summary measures indicated a non-significant difference in executive function of EXPA interventions.

### Memory function

**Figure 5:**
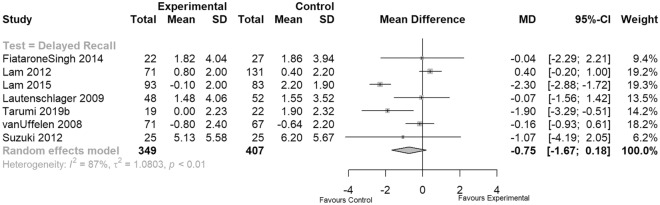
Meta-analysis of memory function.

Memory tests were performed in seven studies and involved both auditory and visual recall test scenarios and included the auditory logical memory I (immediate) and II (delayed) subtests of the Wechsler memory scale third edition (WMS-III)^61^, the list-learning subsection of the ADAS-Cog^51^, visual recall via the benton visual retention test—revised 5th edition (BVRT-R)^62^, the California verbal learning test—second edition (CVLT-II) total score and delayed free recall^63^, and the Consortium to establish a registry for Alzheimer’s disease (CERAD) word list^64^. We pooled the results of seven moderate quality studies reporting 12- or 18-month data on delayed recall tasks (see Fig. 5). The results indicate that in terms of improving and protecting memory function, in two studies EXPA interventions significantly performed worse than control groups that did not involve physical activity (n = 756, MD = – 0.75, 95% CI − 1.67 to 0.18; Fig. 5). The overall result showed no significant difference.

### Attention and speed

**Figure 6:**
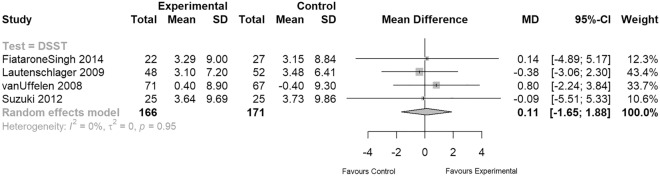
Meta-analysis of attention and speed.

Attention and speed was assessed in six studies using individual digit and visual span tests, as well as the following digit symbol substitution tests: (DSST) Symbol digit modalities test^65^, digit symbol substitution test^66^, and digit symbol coding total^67^. It was possible to conduct a meta-analysis on DSST scores including data from four moderate quality studies (see Fig. 6). The summary measure indicated no significant difference between EXPA intervention and control groups in terms of improving executive function of speed and attention (n = 337, MD = 0.11, 95% CI − 1.65 to 1.88; Fig. 6).

### (Instrumental) activities of daily living

(Instrumental) activities of daily living (I)ADL were measured in four studies with overall moderate quality by the Chinese disability assessment for dementia (CDAD)^68^^,^^45^, by the Timed instrumental activities of daily living (TIADL)^69^^,^^31^, by the Alzheimer’s disease cooperative study-activities of daily living (ADCS-ADL)^70^^,^^32^, and by mental capacity to perform daily tasks by the Bayer activities of daily living (B-IADL)^71^^,^^41^. No significant changes were found in CDAD or TIADL. Small between-group differences were observed in ADCS-ADL, but they were not statistically significant. Functional status with B-IADL improved significantly after 18 months in favor of 6-month progressive resistance training, as compared with a stretching and video watching control group^41^. Since the reported outcome measures of (I)ADL could not be pooled meaningfully, a forest plot was published in the Zenodo repository for open access without a combined analysis of the individual studies^22^.

### Health-related quality of life

Three studies with low-to-moderate quality assessed health-related quality of life. Two studies^39,44^ reported health-related quality of life by using the Medical Outcomes 36-Item Short Form (SF-36) and its even shorter form SF-12^72^ for both the mental and physical component summary. Furthermore, the health-related quality of life for people with dementia scale (DQOL)^73^^,^^44^ and the DEMQOL tool was reported^42,74^. There was no statistically significant effect in favour of EXPA interventions over sham-exercise controls 12 or 18 months after start of intervention. Due to high heterogeneity in terms of outcome measurements only a forest plot was published in the Zenodo repository for open access^22^.

### Healthcare utilization

Healthcare utilization was not an outcome measured in any of the included studies.

### Caregiver outcomes

None of the included studies measured caregiver outcomes.

### Psychosocial functioning

Four low-to-moderate quality studies assessed psychosocial functioning. Three of the included studies^39,45,47^ used the Cornell scale for depression in dementia (CSDD)^75^ and the Beck depression inventory (BDI)^76^ to assess depressive symptoms. After 12 and 18 months, none of the studies showed either EXPA interventions or sham-exercise to have a significant effect on depressive symptoms. One study^31^ reported social functioning based on the Cognitive Self-Report Questionnaire (CSRQ) Social subscale^77^, but did not show significant effects of an interactive video gaming intervention *versus* a health education control group on self-rated social functioning. A forest plot was published in the Zenodo repository for open access^22^.

### Physical functioning

Physical functioning was assessed in six studies with overall moderate quality. Lam et al.^46,47^, used the Berg balance scale (BBS)^78^ to assess functional balance. In an intention-to-treat analysis, the performance of the Tai Chi group was better than that of the stretching and toning exercise group. Peak oxygen consumption measurements (VO2peak) during treadmill stress tests were used in two studies^34,42^, and indicated that after 12 months, aerobic fitness had improved in the aerobic exercise intervention groups compared to the stretching and toning control groups, but the improvement was not significant. Hughes et al.^31^ used gait speed in seconds as a measure of physical functioning, but the results were not significantly different to those in the Wii and the health education control groups. Liu-Ambrose et al.^32,33^ used the physical activities scale for the elderly (PASE) questionnaire^79^ and the 6-min walking test (6MWT)^80^, but were unable to report significant within-group changes or between-group differences in either of the two measures between baseline and month 12. 6MWT was also reported by Uemura et al.^49^, but no significant difference could be demonstrated between combined aerobic, strength and balance training and an educational control group, after 12 months. A forest plot was published in the Zenodo repository for open access^22^.

### Pain

Pain was not included as an outcome in any of the studies in this review.

### Motivational parameters

Only Hughes et al. assessed motivation to participate, which was very high in both intervention and control groups^31^. Self-rated satisfaction as well as mental and social stimulation was very high among the majority of participants. However, the Wii and control groups did not significantly differ in terms of motivation in this study with mostly unclear RoB. No other motivational parameters were included as outcomes in any of the studies in this review.

### Adverse events

Adverse events were assessed in eight studies^32,35,36,39,41–47^. Fiatarone Singh et al.^41^ reported seven adverse musculoskeletal events—three falls, three exacerbations of pre-existing arthritis, and one non-resolved exacerbation of an underlying rotator cuff tear in the strength training group but lacking report of adverse events in other intervention arms. Lautenschlager et al.^39^ reported 10 events across groups that were thought to be unrelated to the intervention with three events having occurred in the active phase of the exercise intervention. Tarumi et al.^34^^,^^35^ reported four adverse events across groups with half of them having occurred in the aerobic training group, including knee and ankle pain, as well as one fall from the treadmill. Liu-Ambrose et al.^32^ reported three study-related non-syncopal falls, of which two occurred in the aerobic training group and the other in the usual care plus education group. Zero adverse events across groups apart from falls unrelated to the exercise intervention occurred in four studies^42–47^.

### Neurobiological outcomes

Neurobiological outcomes were reported in one good quality study in different publications. Tarumi et al.^34^ included measures such as a decrease in brain volume, and Aß plaque deposition in the brain that had increased over time, but did not differentiate between the aerobic training and the stretching and toning groups. In Tomoto et al.^38^, cardiovascular and cerebrovascular hemodynamics were measured using MRI measurements of brain tissue volume and white matter hyperintensity. In the aerobic group, the carotid ß-stiffness index and cerebral blood flow pulsatility decreased, while normalized cerebral blood flow increased, in comparison to the stretching and toning groups; these differences were statistically significant. Twelve months after the initiation of an exercise intervention, Broadhouse et al.^40^ reported the protective effect of high-intensity resistance training on the degeneration of hippocampal structures that are relevant in the pathology of Alzheimer’s Disease.

### Compliance

All studies reported on adherence or attendance. In Fiatarone Singh et al.^41^, no significant group differences were observed regarding the median training duration (26 weeks), or the mean number of sessions (42 sessions). Average exercise compliance in the aerobic training group was 68% in the study by Liu-Ambrose et al.^32^ without having assessed this data for the control group. In Lautenschlager et al.^39^, adherence to the prescribed exercise over the 24-week period was 78%. At 12 and 18 months, a respective 29% and 19% in the physical activity group and 18% and 19% in the usual care group achieved the equivalent of 70,000 steps or more per week (complete-case analysis only). Adherence reported by Suzuki et al.^48^ was 79.2% in the exercise group without assessment of control group compliance. In van Uffelen et al.^43,44^, median adherence of 63% in the walking and the placebo activity programs did not differ between the two groups. Stuckenschneider et al.^42^ reported that 53% of participants reached the prescribed exercise frequency of 100 sessions over 12 months but did not differentiate between exercise and toning groups. Tarumi et al.^34^ reported a drop-out rate of 31% without differentiating between exercise and control group. Hughes et al.^31^ reported good attendance rates of 20 out of 24 sessions in the majority of participants for both the exercise and control group. Drop-out rates reported by Lam et al.^46^ showed that compliance was lower in the Tai Chi group than the stretching control group. Adherence in Lam et al.^45^ was rated as satisfactory, with 75% in the EXPA intervention and 71% in the control group attending social gatherings.

### Consensus on exercise reporting

The studies did not provide sufficient information on the exercise interventions to enable patients and therapists to base exercise recommendations on them and put them into practice (Table 2). The studies particularly lacked information on the employed exercise equipment, the exercise program, and for what reason the decision was made to starting exercising. Furthermore, it was unclear whether participants were expected to exercise at home, whether the programs included relaxation measures, whether individual tailoring took place, and how adherence and treatment compliance were assessed.

**Table 2:**
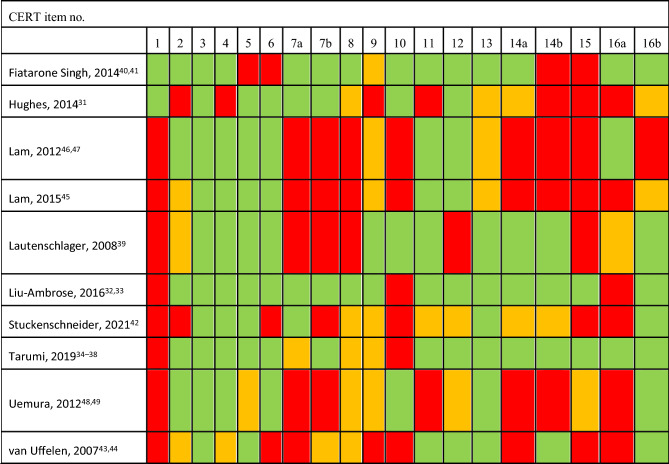
CERT outcomes.

Colour code: Green = Information was reported, Orange = Information was not clearly reported, Red = Information was not reported; Item 1: Detailed description of type of exercise equipment; Item 2: Detailed description of qualifications, expertise and/or training; Item 3: Description whether exercises were performed individually or in a group; Item 4: Description whether exercises were supervised or unsupervised, how they were delivered; Item 5: Detailed description how adherence to exercise was measured and reported; Item 6: Detailed description of motivational strategies; Item 7a: Detailed description of the decision rule(s) for determining exercise progression; Item 7b: Detailed description of how the exercise program was progressed; Item 8: Detailed description of each exercise to enable replication; Item 9: Detailed description of any home program component, Item 10: Information on inclusion of non-exercise components; Item 11: Description of type and number of adverse events that occurred during exercise; Item 12: Description of setting in which exercises were performed; Item 13: Detailed description of exercise intervention; Item 14a: Description whether exercises were generic (one size fits all) or tailored; Item 14b: Detailed description how exercises were tailored to the individual; Item 15: Description of criteria used to determine starting level; Item 16a: Description how adherence or fidelity was assessed/measured; Item 16b: Description of extent to which intervention was delivered as planned.

### Patient preferences

Table 3 provides an overview on the frequencies of outcome parameters reported in studies compared to outcomes preferred by patients based on the initial workshops involving eight patients with MCI. Psychosocial health goals were rated as the most important endpoints for patients. However, freedom from pain, freedom from stress, mood, motivation and self-efficacy were only addressed in one of the studies^31^. No studies were found on either the long-term effects of EXPA programs on the health of relatives or the use of health services. People with MCI also expressed concern that participation in EXPA programs put social pressure on them to stay young and fit. The extent to which EXPA programs adequately addressed this concern in their design and delivery was not described in the studies and should be considered when therapists recommend physical activity and exercise.

**Table 3:** Reported outcomes and outcomes preferred by patients with MCI.

Outcomes	Studies–n (%)	Patient preferences (the more “ + ” the higher the rating)
Adherence	10 (100)	+ +
Executive function	10 (100)	
Global cognition	9 (90)	+
Adverse events	8 (80)	+
Physical fitness	8 (80)	+ +
Memory function	7 (70)	
Speed and attention	6 (60)	
Functional status	4 (40)	
Mood and psychosocial functioning	4 (40)	+ +
Quality of life	3 (30)	
Neuropsychiatric symptoms	1 (10)	+
Progression to dementia	1 (10)	+
Neurophysiological outcomes	1 (10)	
Health care utilization	0 (0)	
Caregiver outcomes	0 (0)	
Pain	0 (0)	
Motivation	0 (0)	+ + +

Table 3 compares the frequencies of outcome parameters reported in studies with those preferred by patients.

## Discussion

This systematic review attempted to determine the effectiveness of EXPA interventions in improving long-term patient- relevant cognitive and non-cognitive outcomes in people with MCI. Ten studies (19 publications) were identified involving 1741 participants with MCI and a mean MMSE of 26, of which most were women (64%), between 65 and 78 years, and not regularly active. The majority of interventions lasted 12 months and involved supervised group-based aerobic exercises tailored to the individual, as well as unsupervised home sessions of moderate intensity and a mean overall exercise volume of 79 h in total.

We examined the effectiveness of EXPA interventions on global cognitive function 12 or 18 months after the initiation of exercise or physical activity in predominately inactive older adults with MCI. Only the meta-analysis combining CDR-SOB data showed a significant improvement while the analyses of ADAS-COG and MMSE showed just a trend or no difference between EXPA interventions and health education or stretching and toning control groups, respectively. This result agrees with other systematic reviews involving short-term interventions and short-term cognitive outcome assessments^81,82^. Therefore, we can assume that positive effects of EXPA on global cognition as measured by CDR-SOB persist beyond the often studied period of up to 6 months. However, it is still unclear whether this long-term effect is valid for previously very active older adults with MCI.

However, Biazus-Sehn et al. showed that physical exercise not only significantly improved global cognitive function, but also executive function and delayed recall^81^. Likewise, Zheng et al. performed a systematic review that compared the effect of aerobic exercise with no specific exercise intervention on cognitive function in older people with MCI. They concluded that aerobic exercise significantly improved global cognitive ability (measured by MMSE and Montreal Cognitive Assessment (MoCA) scores and memory (immediate recall and delay recall), but showed no significant improvements in any other specific domains of cognition^82^. In contrast and with regard to long-term outcomes, we demonstrate no significant difference between EXPA interventions and control groups for global cognition measured by ADAS-COG and MMSE and for domain-specific cognition measured by DSST, VFT, SCWT and delayed recall tasks. Regarding memory function after 12 or 18 months, two studies showed a significant difference favoring control conditions over EXPA interventions. Based on our synthesis including only long-term EXPA interventions, previously published shorter-term effects of EXPA were not evident in our analysis after 12 months or longer assuming no superior effect of EXPA on slowing domain-specific cognitive decline compared to social or educational control conditions. However, we cannot rule out that exercise stimuli plateaued in participants as progression and determination of starting levels was commonly poorly reported in the included studies.

Results for long-term outcomes of IADL, health-related quality of life, psychosocial functioning, and physical fitness could not be pooled because of differences in the employed measures. Hence, evidence on long-term effects of EXPA interventions on non-cognitive health outcomes remain on the level of individual primary studies with inconclusive results and statistically non-significant differences between intervention and control groups.

### Limitations

With an I^2^ of 84%, statistical heterogeneity was considerable in the meta-analysis of CDR-SOB data which reported a significant improvement of global cognition favouring the EXPA intervention. It only included three studies. Concerning patient populations two of these studies recruited Chinese older adults from nursing homes with relatively low levels of education, of which most were women (76–78%)^45–47^ whereas Lautenschlager et al. recruited outpatient Australian older adults with high levels of education of which 51% were women^39^. All of the studies included control groups that were socially active but physically not active including health education sessions, stretching session and tea gatherings or film watching. The mode of EXPA interventions was also comparable across these studies as they all incorporated supervised group-based training as well as unsupervised home-based sessions of moderate intensity. However, the overall exercise dose and stimuli differed between a 48-week Tai Chi training three times a week for 30 min^46,47^, a weekly 60-min multicomponent exercise program for 48 weeks^45^ and a 24-week walking training three times per week for 50 min^39^. Due to poor reporting of intervention details with regard to how the exercise program was progressed, how a starting level was determined or whether the exercise was individually tailored to the participant’s fitness level or development, it remains unclear if those intervention details may have contributed to heterogeneity of effect measures in the CDR-SOB meta-analysis. With regard to the small number of studies in this meta-analysis, subgroup analysis was not suitable. Hence, interpretation need to be made with caution since above variations in intervention and population characteristics may have influenced the result.

Another limitation is that CERT results were extracted by one reviewer only (MD).

### Implications

Although the included cognitive and non-cognitive outcomes EXPA interventions did not show clinically relevant effectiveness when compared to control groups, long-term EXPA interventions (6 + -months) were safe with adherence rates ranging from 53 to 79% of target EXPA duration. However, the studies did not report sufficient information on the exercise interventions to enable patients and therapists to base exercise recommendations on them and put them into practice. Better reporting of details of the interventions could accelerate the translation of research findings into recommendations for EXPA in general practice.

Freedom from pain and stress, mood, motivation and self-efficacy were considered to be important by patients with MCI but were rarely addressed in the studies. A priority in future trials should be to close this research gap. Furthermore, we found no information on the long-term effects of EXPA on the health of relatives, or on the use of health services.

Currently, most long-term EXPA interventions take place in wealthy countries. However, developing countries are a focus of concern because of rising dementia prevalence and associated challenges in the organization of healthcare for people living with dementia^83^. Since EXPA interventions are safe and need not be cost-intensive, healthcare strategies involving physical activity and exercise may help in the secondary prevention of dementia in persons with MCI in countries with limited healthcare resources.

Overall, RoB was introduced by unmasking professionals and using incomplete outcome data. The relationship between RoB and allocation concealment was unclear. In agreement with published guidelines, we found that the overall quality of evidence was low^1,12^. These methodological gaps should be addressed in future trials by carefully planning study design aspects and through compliance with reporting standards.

## Conclusion

In synopsis of the included studies, 6 + -month EXPA interventions tend to improve global cognition 12 months after initiation. Only the meta-analysis of studies with the global cognition scale CDR-SOB demonstrated a significant improvement with considerable heterogeneity, however. Evidence on long-term effects of EXPA interventions on non-cognitive health outcomes could not be meaningfully pooled with the individual studies reporting mixed results that were mostly statistically non-significant. There still exists a research gap in the assessment of non-cognitive health outcomes that are important to patients with MCI, and too little information is available on intervention details for EXPA programs to be replicated and confidently recommended for patients with MCI.

### Supplementary Information

Supplementary Table 1.

## Data Availability

The datasets analysed during the current study are included in this manuscript or available in the Zenodo repository. https://doi.org/10.5281/zenodo.5589276.
