# Supplementary material for: Effectiveness of exercise interventions to improve long-term outcomes in people living with mild cognitive impairment: a systematic review and meta-analysis
Source: Sci Rep. 2023 Oct 23;13:18074. doi: 10.1038/s41598-023-44771-7 (PMC10593841; doi:10.1038/s41598-023-44771-7)
Supplement: Supplementary file 1 — Supplementary Table 1. [file 41598_2023_44771_MOESM1_ESM.docx]

| **Index author, year (refer­ence)** | **Country** | **Setting** | **Design** | **Duration (weeks)** | **Participants, n baseline (% Male)** | **Age, mean (SD)** | **Educational level (years), mean (SD)** | **MMSE score, mean (SD)** | **Fitness level at baseline** | **Relevant interventions, overall exercise volume in min (min x d x wks)** | **Exercise modalities** | **Outcomes** |
| --- | --- | --- | --- | --- | --- | --- | --- | --- | --- | --- | --- | --- |
| Fiatarone Singh, 2014 [33, 34] | Australia | Community-based | Fully factorial design (2x2) | 72 | 100 (49) | 74 (nr) | nr | 27 (2) | Participants that exercised >150 min/wk on a regular basis were excluded | IG: Progressive resistance training 3600 min  CG: Video watching plus stretching exercises | 75 min/day, 2 days/week, 24 weeks, high intensity, supervised, group-based, individually tailored exercise | Global cognition; Executive action; Memory function, Speed/attention; Functional status; Physical fitness; Adherence; Adverse events; Neurophysiological measures |
| Hughes, 2014 [24] | USA | Outpatient (hospital) | Pilot, Parallel arms | 48 | 20 (70) | 77 (6) | 14 (2) | 27 (2) | nr | IG: Wii interactive video gaming, 2160 min  CG: Health education | 90 min/day, 1 day/week, 24 weeks, exercise intensity nr, face-to-face, (un-) supervised nr, group-based, one-size-fits-all exercise | Global cognition; Executive action; Mood and Psychosocial functioning; Functional status; Physical fitness; Adherence |
| Lam, 2012 [39, 40] | China | Long-term care centers and nursing homes; Community-based | Cluster | 48 | 389 (24) | 78 (7) | 3 (4) | 25 (3) | Did not previously practice Tai Chi or other mind-body-exercise regularly for more than 6 months | IG: Tai Chi, 4320 min  CG: Stretching & toning | 30 min/day, 3 days/week, 48 weeks, moderate intensity, supervised in groups or individually and home-based with no supervision, tailoring nr | Global cognition; Executive action; Memory function; Speed/Attention; Physical fitness; Mood; Progression to dementia; Neuropsychiatric symptoms; Adverse events; Adherence |
| Lam, 2015 [38] | China | Long-term care centers and nursing homes | Cluster | 48 | 555 (22) | 75 (7) | 4 (4) | 26 (2) | nr | IG: Aerobic exercise, mind body exercise, resistance training, stretching and toning (e.g. cycling, brisk walking, Tai Chi), 2880 min  CG: Social activities (e.g. tea gathering, film watching) | 60 min/day, 1 day/week, 48 weeks, moderate intensity, supervised group-based and unsupervised home-based, tailoring nr | Global cognition; Executive action; Memory function; Speed/Attention; Functional status; Adverse events; Mood; Adherence |
| Lautenschlager, 2008 [32] | Australia | Outpatient (hospital) | Parallel arms | 72 | 170 (49) | 69 (9) | 12 (3) | nr | 25-33% of participants were previously physically active (70,000 steps/wk), others inactive | IG: Aerobic physical activity (predominantly walking, 3600 min  CG: Health education | 50 min/day, 3 days/week, 24 weeks, moderate intensity, unsupervised home-based or supervised group-based, individual tailoring | Global cognition; Executive action; Memory function; Speed/Attention; Physical fitness; Mood; Quality of life; Adverse events |
| Liu-Ambrose, 2016 [25, 26] | USA | Outpatient (hospital) | Pilot, Parallel arms | 48 | 70 (49) | 74 (8) | nr | nr | Physical activity scale for the elderly indicates age-appropriate activity levels | IG: Aerobic training (Walking), 4320 min  CG: Health Education | 60 min/day, 3 days/week, 24 weeks, progressive intensity, supervised group-based, individually tailored intensity | Global cognition; Executive function; Speed/Attention ; Functional status; Physical fitness; Adverse events; Adherence |
| Stuckenschneider, 2021 [35] | Germany, Ireland, The Netherlands | Outpatient (hospital), Community-based | Parallel arms | 48 | 183 (51) | 72 (7) | nr | nr | Previously sedentary | IG: Aerobic exercise (indoor and outdoor walking and running), 6480 min  CG: Stretching & toning | 45 min/day, 3 days/week, 48 weeks, moderate intensity, Supervised group-based or individual home-based, individually tailored | Global cognition; Executive function; Physical fitness; Quality of life; Adverse events; Adherence |
| Tarumi, 2019 [27–31] | USA | Outpatient care (hospital); Long-term care centers and nursing homes | Parallel arms | 48 | 70 (24) | 65 (6) | 16 (2) | 29 (1) | Participants that exercised regularly were excluded | IG: Aerobic exercise, 5460 min  CG: Stretching & toning | 25-40 min/day, 3-4/week, 48 weeks, high intensity, supervised or unsupervised, individually tailored intensity and progression | Executive action; Memory function; Physical fitness; Adverse events; Adherence; Neurophysiological measures |
| Uemura, 2012 [41, 42] | Japan | Community-based | Parallel arms | 48 | 100 (51) | 75 (7) | 11 (3) | 27 (3) | nr | IG: combined aerobic, strength and balance training 8640 min  CG: Health Education | 90 min/day, 2 days/ week, 48 weeks, moderate intensity, supervised or unsupervised group-based, tailoring nr | Global cognition; Executive action; Memory function; Speed/Attention Physical fitness; Adherence |
| van Uffelen, 2007 [36, 37] | The Netherlands | Community-based | Fully factorial design (2x2) | 48 | 152 (56) | 75 (3) | nr | 29 (1) | Participants were physically active (>3 MET) an average of 39-44 min/d | IG: Walking, 5760 min  CG: low-intensity placebo activity program | 60 min/day, 2 days/week, 48 weeks, moderate intensity, supervised group-based, individually tailored intensity | Global cognition; Executive action; Memory function; Speed/Attention; Adverse events; Adherence; Quality of life |
